# Effect of Dual‐Site Non‐Invasive Brain Stimulation on Upper‐Limb Function After Stroke: A Systematic Review and Meta‐Analysis

**DOI:** 10.1002/brb3.70145

**Published:** 2024-11-07

**Authors:** Meng Ren, Jingjing Xu, Wenjing Wang, Lexian Shen, Chaojie Wang, Haoyang Liu, Lu Chen, Chanjing Liu, Yongheng Tang, Jiening Wang, Tiantian Liu

**Affiliations:** ^1^ RainbowFish Rehabilitation and Nursing School Hangzhou Vocational and Technical College Hangzhou China; ^2^ School of Rehabilitation Science Shanghai University of Traditional Chinese Medicine Shanghai China; ^3^ Athletic rehabilitation Teaching and Research Office, School of Exercise and Health Guangzhou Sport University Guangzhou China; ^4^ Seventh People's Hospital Affiliated to Shanghai University of Traditional Chinese Medicine Shanghai China; ^5^ School of Foreign Languages Shanghai Jiao Tong University Shanghai China; ^6^ Acupuncture and Moxibustion Massage College Liaoning University of Traditional Chinese Medicine Shenyang China

**Keywords:** meta‐analysis, stroke, transcranial direct current stimulation, transcranial magnetic stimulation, upper extremity

## Abstract

**Background:**

Non‐invasive brain stimulation (NIBS) has attracted significant attention as it has been proven to be effective in facilitating upper limb motor recovery in patients with stroke. This meta‐analysis evaluates the efficacy of dual‐site non‐invasive brain stimulation (DS‐NIBS) in improving upper extremity motor function after stroke.

**Methods:**

A PRISMA systematic search was conducted for randomized controlled trials. Two authors independently extracted data, and the quality of included studies was assessed.

**Results:**

Ten studies were included in the current review. DS‐NIBS demonstrated a significant effect on upper extremity motor function impairment. However, only two studies showed no clear effects of DS‐tDCS on upper extremity motor function after stroke. Due to the limited number of studies, the effects of DS‐NIBS remain inconclusive.

**Finding:**

This review found evidence for the relatively higher efficacy of DS‐NIBS on post‐stroke upper extremity motor function impairment, compared to the sham and SS‐NIBS. Additionally, DS‐TMS was found to generate better improvement than DS‐tDCS.

## Graphical abstract

This meta‐analysis evaluates the efficacy of dual‐site non‐invasive brain stimulation (DS‐NIBS) in improving upper extremity motor function after stroke. The analysis included 10 trials with 426 participants, indicating that DS‐NIBS showed higher efficacy on post‐stroke upper extremity motor function impairment compared to the sham and single‐NIBS. Additionally, dual‐site repetitive transcranial magnetic stimulation was found to generate better improvement than dual‐site transcranial direct current stimulation.

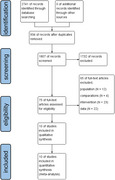

## 1 Introduction

Stroke is one of the most important challenges faced by the world medical community, being a leading cause of death and disability (Collaborators 2021; Owolabi et al. 2022; Stinear et al. 2020). Hemiplegia is the most common functional disability after stroke, and upper limb hemiplegia (including hand dysfunction) has always been an important and difficult problem in stroke rehabilitation, affecting daily living and increasing the burden on these patients and their families (Lawrence et al. 2001; Min and Min 2015; Vlisides and Mashour 2016). Conventional rehabilitation training techniques for upper limb dysfunction are limited, including occupational therapy, neurostimulation techniques, strength training, motor relearning, restriction‐induced motor training, and so on (Gittler and Davis 2018; Langhorne, Bernhardt, and Kwakkel 2011), and often show variable and limited effectiveness (Barreca et al. 2003).

Various patterns of neural network reorganization occur in both the lesioned and unaffected hemispheres after stroke (Cramer 2008), and functional recovery is associated with neural plastic changes in the brain (Volz et al. 2016), including neurogenesis, gliogenesis, axonal sprouting, changes in excitation/inhibition balance, and many more. The relationship between stroke motor recovery and cortical reorganization has been explored by many scientists (Sinke et al. 2018; Vidal et al. 2018; Xerri et al. 2014), revealing the importance of the inter‐hemispheric activation balance in motor‐related cortices for motor recovery of stroke patients (Tang et al. 2015). Therefore, cortical excitability regulation has become a therapeutic strategy for stroke.

Non‐invasive brain stimulation (NIBS) entails the modulation of brain excitability and activity (Caglayan et al. 2019; Cheng et al. 2023). At the cellular level, NIBS is capable of enhancing cellular neuromodulation. This modulation includes the reduction of the inflammatory response, autophagy suppression, antiapoptotic effects, angiogenesis enhancement, alterations in the blood‐brain barrier permeability, attenuation of oxidative stress, influence on neurotransmitter metabolism, neurogenesis, and enhanced structural neuroplasticity (Badoiu et al. 2023; Ferreira et al. 2024). Transcranial direct current stimulation (tDCS) and transcranial magnetic stimulation (TMS) were considered the two most common methods used for NIBS (Li et al. 2023). Existing evidence suggests that tDCS can elicit either an excitatory or inhibitory effect. More specifically, anodal tDCS can increase the function of the targeted cortical areas, whereas cathodal tDCS elicits a suppressive effect (Fertonani and Miniussi 2017). These effects were commonly modulated through Ca2+ signaling. In synapses, Ca2+ influxes into the postsynaptic cell through activating AMPA‐subtype glutamate receptors to trigger synapse plasticity, such as long‐term potentiation and long‐term depression, to improve neuroplasticity (Shepherd and Huganir 2007). Similarly, repetitive transcranial magnetic stimulation (rTMS) has dual effects of facilitation (high frequency, > 1 Hz, commonly used > 5 Hz) or inhibition (low frequency, ≤ 1 Hz, commonly used 1 Hz) on the excitability of the cerebral cortex and has been widely used in the movement disorders caused by stroke (Bai, Zhang, and Fong 2022; Edwards et al. 2021).

However, although there are many reports and related guidelines on the application of NIBS for stroke hemiplegia rehabilitation, there is still no consensus on the target, mode, or strategy for stimulation (Khedr et al. 2013; Kubis 2016; Peruzzotti‐Jametti et al. 2013; Wong et al. 2022). This may be related to the degree of brain damage, the course of the disease, the patient's response to interventions, and so on (Boddington and Reynolds 2017).

The concept of interhemispheric competition is the mainstream theory behind neural regulation. Stroke causes damage to one hemisphere, thereby reducing the ipsilateral hemisphere's ability to inhibit the contralateral hemisphere. As a result, the contralateral hemisphere increases inhibition on the affected hemisphere, resulting in an imbalance between the two hemispheres and affecting functional recovery. The Vicariation model complements interhemispheric competition, but some strategies are diametrically opposed. It believes that the lesion of the stroke causes dysfunction, and the over‐activation of both the peri‐lesional brain area and the contralateral hemisphere may be not a maladaptive but rather a vicarious mechanism. Interhemispheric competition and vicariation model have two diametrically opposite directions on whether the contralateral hemisphere cortex is inhibited or excited, causing difficulty for clinicians in choosing neuromodulation strategies for stroke hemiplegia rehabilitation. The bimodal balance‐recovery model moderates some differences between the two models above and introduces the concept of “structural reserve” (Pino et al. 2014). However, it remains controversial what constitutes high or low structural retention.

To achieve better clinical outcomes, some studies also have used dual‐site NIBS for upper extremity motor dysfunction after stroke, focusing on brain circuits (Achacheluee et al. 2018; Cho et al. 2017). Cortico‐cortical paired associative stimulation (ccPAS), which applies repeated pairing of TMS pulses over two distinct cortical sites at precise interpulse intervals, can more effectively alter the excitability of the motor cortex, resulting in better recovery of the upper limb function (Duan et al. 2022). Moreover, cerebello‐motor PAS has also been found effective compared to sham in improving hand dexterity but not grip strength (Rosso et al. 2022). Some researchers also explore the effect of dual‐site tDCS. In one study (Lei et al. 2023), the investigators set up a new method of tDCS in which the cathodal electrode is placed on the ischemic hemisphere and the anodal electrode on the contralateral hemisphere of rats subjected to ischemia‐reperfusion injury. This new method protected against neuronal death and improved the functional recovery of stroke animals, suggesting a potential endogenous therapy. A meta‐analysis on 657 stroke patients demonstrated that bilateral transcranial electric stimulation and cathodal tDCS over the contralesional hemisphere were superior to other stimulation montages/patterns/protocols on gait, balance and/or lower limb motor recovery in stroke patients (Veldema and Gharabaghi 2022).

The present systematic review and meta‐analysis of randomized controlled trials aimed to explore the effects of dual‐site NIBS on the upper limb (UL) motor impairments and functional performance post‐stroke. The secondary goals included investigating whether the NIBS types enhance motor recovery, identifying factors that may contribute to better motor outcomes, and exploring potential adverse effects of using dual‐site NIBS in patients with stroke.

## 2 Methods

### 2.1 Search Strategy

This systematic review and meta‐analysis is reported in accordance with the Preferred Reporting Items for Systematic Reviews and Meta‐Analyses (PRISMA) Statement and was registered at the International Prospective Register of Systematic Reviews (number CRD42022370564). The literature research was conducted in MEDLINE (via PubMed), Cochrane Library, Embase, Web of Science, China National Knowledge Infrastructure (CNKI), WeiPu, WanFang, and the Chinese Biomedical Literature Database (CBM) up to September 30, 2022, without language restriction.

The following keywords were used: stroke, upper extremity, transcranial direct current stimulation, and transcranial magnetic current stimulation. The full search strategy can be found in Supplementary Material (SM), Table SM1.

### 2.2 Study Selection

The inclusion criteria to identify for qualifying articles were:

- randomized control trials (RCTs) that included post‐stroke adult participants (≥ 18 years);
- the focus was on the effects on the upper limb in post stroke patients;
- the types of intervention were dual‐site NIBS;
- the control group included single‐site or sham NIBS;
- reported at least one standardized outcome measure that evaluated the upper‐limb performance, ICF body structure/body function domain (e.g., Fugl‐Meyer Assessment Scale (FMA), Ashworth or modified Ashworth Scale, force and range of motion) or activity level (e.g., Wolf Motor Function Test (WMFT), Action Research Arm Test (ARAT), Box and Blocks Test (BBT), Jebsen‐Taylor Hand Function Test (JTT), Nine Hole Peg Test, and Motor Assessment Scale).

Exclusion criteria were as follows:

- failure to provide relevant data on the outcome measures;
- the dual‐site NIBS was used not only in one group;
- NIBS was applied in combination with other techniques (i.e., use of virtual reality or electrostimulation in adjunction to single‐site NIBS intervention);
- central‐peripheral paired associative stimulation;
- poor quality RCTs (PEDro < 5) were also excluded (This is discussed in detail in Quality Assessment section).

After searching, duplicate records were excluded. Two independent investigators reviewed study titles and abstracts, and studies that satisfied the inclusion criteria were retrieved for full‐text assessment. Two reviewers independently identified the eligible studies according to the pre‐formulated inclusion and exclusion criteria. If any discrepancies arose, a third investigator was consulted and made the final decision.

### 2.3 Data Extraction

Two authors independently extracted data from the included studies. If any discrepancies arose, a third investigator was consulted and made the final decision. If information was missing or unclear in articles, their authors were contacted. For crossover studies, only data from the first intervention were extracted for meta‐analyses.

When multiple outcomes were assessed to measure body function or structure, the Fugl‐Meyer assessment was considered the priority of analysis according to the recommendations of the measurement working group of the Stroke Recovery and Rehabilitation Round table. To measure activity limitation, the ARAT was considered the priority of analysis according to recommendations of the same panel. If these scales were not present in the study, the most frequent outcomes across the selected studies were chosen.

An electronic data extraction form was used to collect the following information: study design, number of subjects, sample characteristics (i.e., mean age, gender, stroke duration, lesion side, and phase), treatment protocol (i.e., frequency, intensity, number of pulses, the time of intervention, the number of sessions, and the target), and outcome measures. In instances where results were only presented in figures and the authors did not report further information despite attempts to contact them, a Plot Digitizer program was used to extract values. This program digitizes uploaded figures by calibrating the image's axes, allowing data points to be extracted by clicking on any data point on the figure. If a study did not report sufficient quantifiable results and the authors did not respond to requests, then the study was excluded.

### 2.4 Quality Assessment

The Physiotherapy Evidence Database (PEDro) scale was used to assess the methodological quality of each included RCT by two independent reviewers. The PEDro scale is an 11‐item scale that has been widely used for rating the methodological quality of RCTs (Baniqued et al. 2021; Brunt, Albines, and Hopkins‐Rosseel 2019). Each satisfied item pertained to internal validity received one point, except the first item, which is rated as YES or NO (maximum score = 10 points). Studies scoring four or higher on the PEDro scale were considered of sufficient quality (Foley et al. 2003). Studies scoring 6 or higher in which the critical criteria 2 or 3 (randomization and concealment of allocation, respectively) were absent were downgraded to fair quality. Poor‐quality studies (scores lower than 4) were excluded in the present study.

### 2.5 Statistical Analysis

For studies using the same scale to evaluate the outcome (i.e., Fugl‐Meyer for body structure/function), the number of participants in each group, mean scores, and SDs after interventions in the active and control groups were analyzed in RevMan 5.3. (Review Manager 5.3). For the Fugl‐Meyer, a higher score was regarded as positive.

For types of outcomes assessed with different scales (i.e., activity limitation), measures of postintervention and preintervention of each subject were assessed after contacting the authors and requesting individual data. The mean and SD of the change scores relative to the baseline or the posttreatment mean and SD were recorded for each outcome measure in the experimental and control groups. For scales in which a lower score was regarded as positive compared with a higher score, the mean was multiplied by −1. The means and SDs of relative differences in active and control groups were analyzed in RevMan 5.3. Regarding the continuous outcomes, if the unit of measurement was consistent across trials, the results were presented as the weighted mean difference (MD) with 95% confidence intervals (95% CIs). Standardized mean differences (SMDs), instead of the mean difference (MD), with 95% confidence intervals (95% CIs), were used if the outcome measurement scale was not identical between studies.

Funnel plots were used to detect publication bias if more than 10 articles were involved in the meta‐analysis.

### 2.6 Additional Analysis

To identify potential influencing factors on motor recovery, subgroup analyses were also performed based on NIBS types (tDCS versus rTMS), post‐stroke duration (acute [⩽ 1 week] versus subacute [1 week to 6 months] versus chronic [> 6 months]), targeted region, theoretical model, lesion location (subcortical versus nonspecified), and treatment sessions. If two or fewer studies were identified for a single analysis objective, only qualitative description instead of meta‐analysis would be performed.

Heterogeneity between studies was assessed using the Cochran Q test and the Higgins’ I^2^ statistic. An I^2^ value below 20% was considered to indicate low levels of heterogeneity, while an I^2^ value above 50% indicated high levels of heterogeneity. A fixed effects model was applied if I^2^ < 50%; otherwise, a random‐effects model would be used.

Publication bias was evaluated using Egger's linear regression test and visual inspection of the funnel plot. Sensitivity analysis was conducted to explore the impact on the effect size when low‐quality studies and studies with cross‐over design were excluded. The level of significance was set at *p* < 0.05 for all statistical analyses. Stata (version 16.0) was used for Egger's linear regression test. Statistical significance was set at two‐tailed *p* < 0.05. Finally, effect sizes were classified as small (0.2), medium (0.2–0.8), and large (0.8).

## 3 Results

### 3.1 Study Characteristics

**FIGURE 1:**
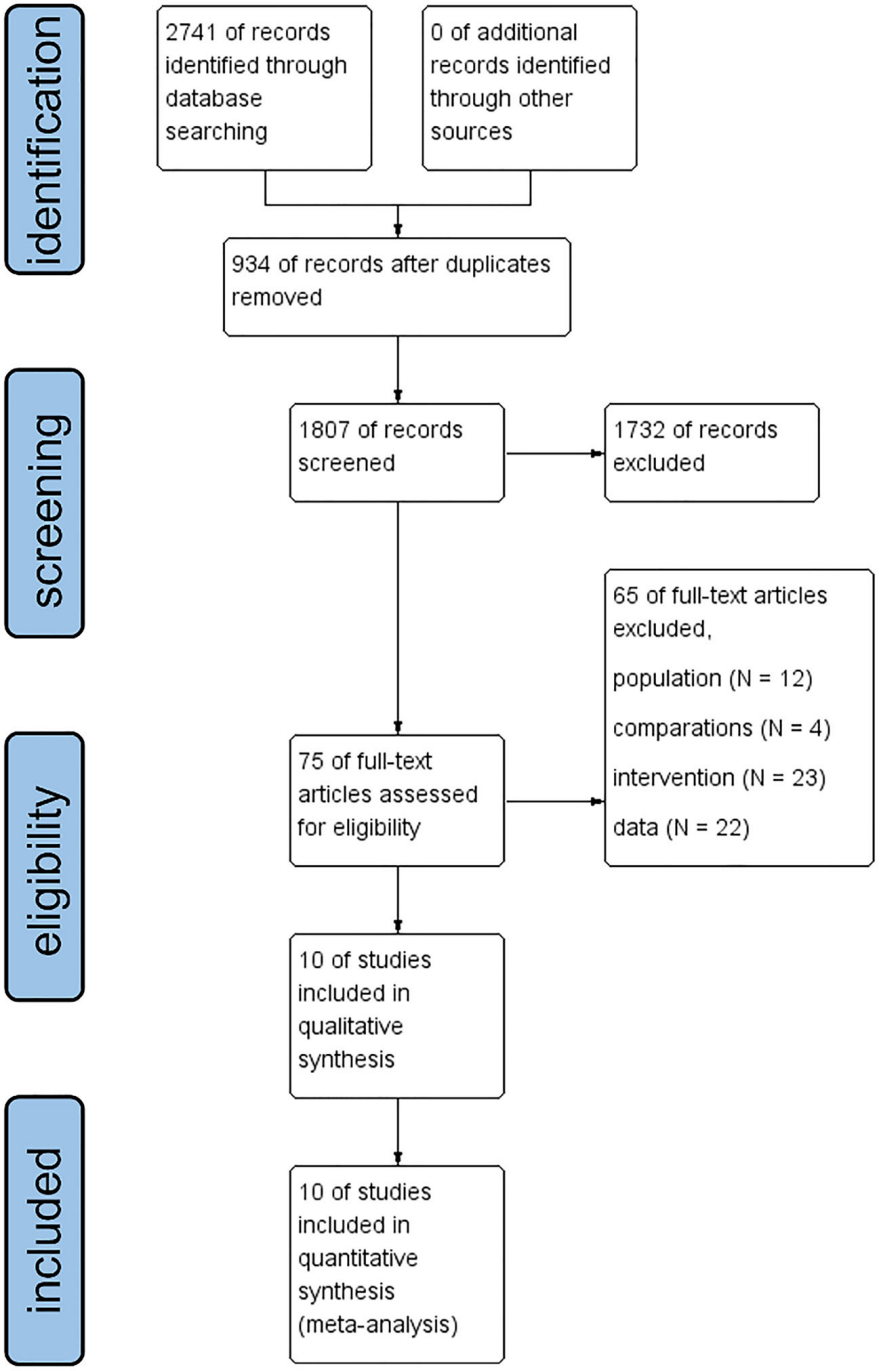
PRISMA Flow Chart diagram.

The initial database search yielded a total of 2,741 relevant studies. Only 10 studies were identified (*N* = 426) by two independent reviewers based on the inclusion criteria. The flow diagram of the selection process is shown in Figure 1.

The characteristics of recruited participants included in this meta‐analysis are detailed in Table 1. All relevant information regarding the studies that meet the inclusion criteria is presented in Table 2. The study protocols contained NIBS with various conditions. In four studies (Hsu et al. 2021; Ji 2014; Kim 2021; Lindenberg et al. 2010), the control group received sham NIBS. In another four studies (Cai 2020; Cao et al. 2022; Fleming et al. 2017; Ren et al. 2018), the control group received single‐site NIBS. The remaining two studies (Long et al. 2018; Taud et al. 2021) contain both sham and single‐site NIBS groups.

**TABLE 1:** Characteristics of recruited participants included in this meta‐analysis.

Reference	Total N	Age (year) [Mean (SD)]	Gender [Male (%)]	Time post stroke [Mean (SD)]	Lesion side [right (%)]	phase
Fleming et al. (2017)	25	DS: 59.8 (13.1) SS: 59.8 (13.1)	NR	DS: 19.7 (27.4) m SS: 19.7 (27.4) m	DS: 12(48%) SS: 12(48%)	chronic
Long et al. (2018)	62	DS: 55.90 (8.89) SS: 57 (11.78) Sham:56.85(5.48)	DS: 16 (76%) SS: 16 (76%) Sham: 15(75%)	DS: 19.81 (2.98) d SS: 19.57 (2.34) d Sham: 19.05(2.74)	DS: 11(52%) SS: 11(52%) Sham: 11(55%)	subacute
Taud et al. (2021)	30	DS: 58.3 (12.8) SS: 60.3 (10.3)	DS: 11 (73%) SS: 12 (80%)	DS: 21.9 (17.2) m SS: 28.8 (35.3) m	DS: 8(53%) SS: 8(53%)	chronic
Cai (2020)	103	DS: 55.37 (8.16) SS: 55.00 (7.55)	DS: 29 (56%) SS: 27 (53%)	DS: 60.31 (16.94) d SS: 61.39 (15.49) d	DS: 26(50%) SS: 27(53%)	subacute
Cao et al. (2022)	40	DS: 51.80 (15.45) SS: 48.75 (13.24)	DS: 12 (60%) SS: 11 (55%)	DS: 45.85 (20.56) d SS: 41.90 (24.86) d	DS: 12(60%) SS: 7(35%)	subacute
Ren et al. (2018)	60	DS: 51.2(3.6) SS:49.6 (4.3)	DS: 19 (63%) SS: 17 (57%)	NR	NR	NR
Hsu et al. (2021)	27	DS: 59.1 (11.4) Sham: 59.2 (11.8)	DS: 9 (69%) Sham: 6 (43%)	DS: 20.7 (3.5) d Sham: 21.1 (5.3)d	DS: 9 (69%) Sham: 7 (50%)	subacute
Kim (2021)	30	DS: 60.2(5.3) Sham: 60.33(6.33)	DS: 7 (47%) Sham: 8 (53%)	DS: 12.13(1.84)m Sham: 10.93(1.94)m	DS: 7 (47%) Sham: 8 (53%)	chronic
Lindenberg et al. (2010)	20	DS: 61.7 (14.7) Sham: 55.8(12.9)	DS: 8 (80%) Sham: 7 (70%)	DS: 30.5(21.4)m Sham: 40.3(23.4)m	DS: 4 (40%) Sham: 3 (30%)	chronic
Ji (2014)	29	DS: 64.2(11.9) Sham: 62.3(12.2)	DS: 11 (73%) Sham: 10(71%)	DS: 8.1 (1.5) m Sham: 8.2 (1.6)m	NR	chronic

Abbreviations: DS: dual‐site NIBS; NR: not reported; SS: single‐site NIBS.

**TABLE 2:** Characteristics of the included studies in this meta‐analysis.

Reference	Type of NIBS	DS intervention group	Comparation group	Outcome measure
Fleming et al. (2017)	tDCS	The anode was placed over ipsilesional M1 and the cathode over contralesional M1.	Anodal tDCS: anodal to ipsilesional M1 and the cathode over the contralateral supraorbital ridge, 1 mA 0.04 mA/cm^2^ Cathodal tDCS: cathodal to contralesional M1 the anode was placed over ipsilesional M1 and the cathode over contralesional M1. Sham	JTT
Long et al. (2018)	TMS	1 Hz rTMS to the cM1 followed by 10 Hz rTMS to the iM1 With an interstimulus interval of 50 seconds. 90% RMT, 1000 pulses, 15 sessions	LF‐rTMS: cM1, 1 Hz, 90% RMT, 1000 pulses, 15 sessions Sham: The coil was held at an angle of 90° to the scalp	FMA‐UL WMFT
Taud et al. (2021)	tDCS	Active anode placed over the iM1 and a smaller active cathode was placed over the cM1. 0.03 mA/cm^2^,23 min, 5 sessions	Anodal tDCS: active anode placed over the iM1 and the cathode was placed over the contralesional supraorbital ridge. Sham: the electrode set‐up was pseudo‐randomly assigned to participants (either anodal or dual) and balanced across the group.	FMA‐UL WMFT
Cai (2020)	TMS	cM1, 1 Hz, 90% RMT, 1000 pulse, 24 sessions and iM1, 10 Hz, 90% RMT, 1000 pulses, 24 sessions	LF‐rTMS: cM1, 1Hz, 90% RMT, 1000 pulses, 24 sessions HF‐rTMS: iM1, 10 Hz,90% RMT, 1000 pulses, 24 sessions	FMA‐UL
Cao et al. (2022)	TMS	iTBS applied to contralateral cerebellar cortex and iM1 separately, an interval of 5s was set between these two stimulations. iTBS: 80%∼100% RMT, repetitive bursts of 3 stimuli at a frequency of 50 Hz repeated at 5 Hz,600 pulses, 24 sessions	iTBS: iM1, 80%∼100% RMT, 600 pulses, 24 sessions	FMA‐UL ARAT
Ren et al. (2018)	TMS	cM1,80%RMT, 1 Hz, 3000 pulses, 14 sessions and iM1,80%RMT, 5 Hz, 3000 pulses, 14 sessions	iM1, 80%RMT, 5 Hz, 3000 pulses, 14 sessions	FMA‐UL
Hsu et al. (2021)	tDCS	iM1 anode and cM1 cathode, 0.08 mA/cm^2^, 20 min, 20 sessions	Sham: tDCS settings were similar except that the direct current ceased after 2 min.	FMA‐UL ARAT
Kim (2021)	tDCS	iM1 anodal electrode, and the cM1 cathodal electrode, 1 mA, 20 min, 20 sessions	Sham: no current flows	FMA‐UL
Lindenberg et al. (2010)	tDCS	Anodal tDCS to iM1 and cathodal tDCS to cM1, 0.09 mA/cm^2^, 30 min, 5 sessions	Sham: The current was ramped up to 1.5 mA and slowly decreased over 30 seconds	FMA‐UL WMFT
Ji (2014)	TMS	cM1, 1 Hz, 80–100%RMT, 900 pulses and iM1, iTBS, 80–120%RMT, 15 min, 30 sessions	Sham: The coil was held at an angle of 90° to the scalp	FMA‐UL

Abbreviations: ARAT: Action Research Arm Test; FMA‐UL: Fugl‐Meyer Assessment upper limb; JTT: Jebsen‐Taylor Hand Function Test; LF‐rTMS: low‐frequency repetitive transcranial magnetic stimulation; tDCS: transcranial direct current stimulation; WMFT: Wolf Motor Function Test.

The study intervention contained various NIBS. Five studies (Fleming et al. 2017; Hsu et al. 2021; Klomjai et al. 2022; Lindenberg et al. 2010; Taud et al. 2021) applied tDCS, while five studies (Cai 2020; Cao et al. 2022; Ji 2014; Long et al. 2018; Ren et al. 2018) applied rTMS. According to the outcome measures of upper limb function, nine studies reported FMA‐UL, while only one study (Fleming et al. 2017) reported JTT.

### 3.2 Adverse Effects

All participants tolerated DS‐NIBS without significant adverse events. No adverse effects were observed by the investigators or reported by the DS‐NIBS patients.

### 3.3 Quality Assessment

Table 3 presents the methodological quality assessment of the included studies, as evaluated using the PEDro scale. All of the included studies scored more than 4 points on the PEDro scale, indicating sufficient quality. The mean score of PEDro was 6.7 points (SD = 0.82), ranging from 5 to 8 points.

**TABLE 3:** Methodological quality of included studies.

Reference	Eligibility criteria specified (Yes/No)	Random allocation (0/1)	Concealed allocation (0/1)	Comparable at baseline (0/1)	Blinded subjects (0/1)	Blinded therapists (0/1)	Blinded assessors (0/1)	Adequate follow‐up (0/1)	Intention‐totreat analysis (0/1)	Between group comparisons (0/1)	Point estimates and variability (0/1)	Summary
Fleming et al. (2017)	Yes	1	1	0	1	0	0	1	1	1	1	7
Long et al. (2018)	Yes	1	0	1	1	0	1	1	1	1	1	8
Taud et al. (2021)	Yes	1	0	1	1	0	1	1	0	1	1	7
Cai (2020)	Yes	1	0	1	1	0	1	1	0	1	1	7
Cao et al. (2022)	Yes	1	0	1	0	0	1	1	0	1	1	6
Ren et al. (2018)	Yes	1	0	1	0	0	0	1	0	1	1	5
Hsu et al. (2021)	Yes	0	1	1	1	0	1	1	0	1	1	7
Kim (2021)	Yes	1	0	1	1	0	1	1	0	1	1	7
Lindenberg et al. (2010)	Yes	0	0	1	1	1	0	1	0	1	1	6
Ji (2014)	Yes	1	0	1	1	0	1	1	0	1	1	7

### 3.4 Meta‐Analysis Results

**FIGURE 2:**
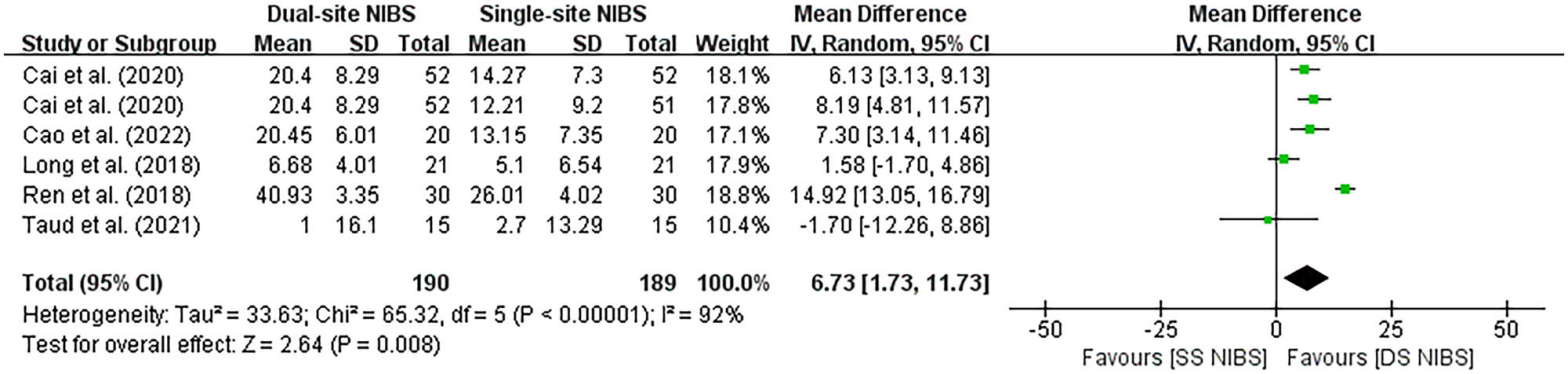
Forest plot of DS‐NIBS versus SS‐NIBS. Results of meta‐analysis comparing the FMA‐UL of DS‐NIBS versus SS‐NIBS in treating upper extremity motor function after stroke. FMA‐UL: Fugl‐Meyer Assessment upper limb; DS‐NIBS: dual‐site non‐invasive brain stimulation; SS‐NIBS: single‐site non‐invasive brain stimulation.

The effect of dual‐site NIBS on upper limb function after stroke compared with sham and single‐site NIBS was evaluated by pooling post‐intervention data from 10 studies involving 426 participants. The pooled meta‐analysis showed a significant improvement on Fugl‐Meyer Assessment upper limb (FMA‐UL) scores after dual‐site NIBS in the clinical population (*p* = 0.008), compared with SS‐NIBS (Figure 2).

Dual‐site NIBS was significantly more effective than sham simulation (MD, 3.18; 95% CI, 1.73 to 4.63) and single‐site NIBS (MD, 9.65; 95% CI, 8.4 to 10.89) with respect to motor function, respectively. However, significant evidence of inter‐study heterogeneity was observed for the meta‐analysis in sham simulation and single‐site NIBS (I^2^ = 44%, *p* < 0.0001 and I^2^ = 92%, *p* = 0.008, respectively).

**FIGURE 3:**
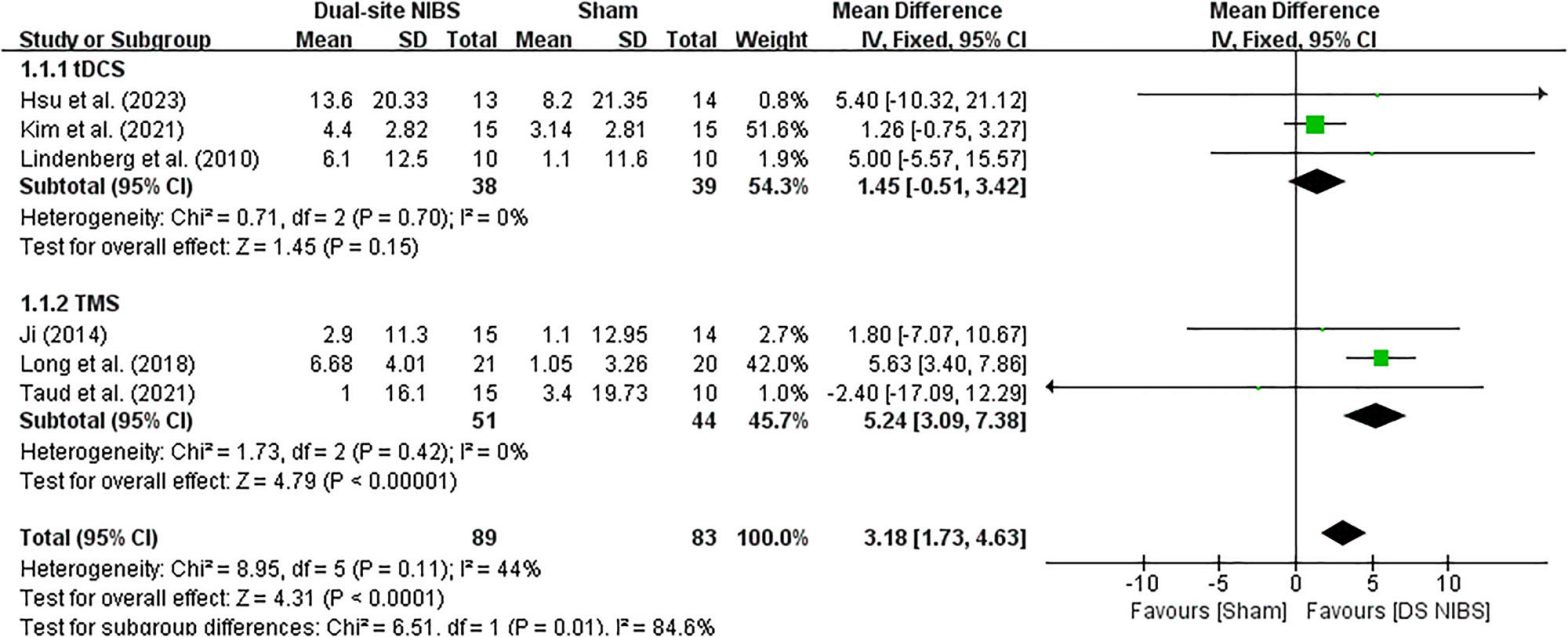
Forest plot of DS‐NIBS versus Sham‐NIBS. Results of subgroup analysis comparing the FMA‐UL of DS‐NIBS versus Sham‐NIBS in treating upper extremity motor function after stroke. FMA‐UL: Fugl‐Meyer Assessment upper limb; DS‐NIBS: dual‐site non‐invasive brain stimulation; SS‐NIBS: single‐site non‐invasive brain stimulation.

A further subgroup meta‐analysis was conducted according to the different NIBS types. Because there are only two studies using tDCS for DS‐NIBS versus SS‐NIBS, the subgroup meta‐analysis was only performed for DS‐NIBS versus Sham‐NIBS. When considering the three DS‐tDCS trials alone, there was a moderate but non‐significant pooled effect size (0.52, *p* = 0.15) favoring the stimulation intervention. The three trials investigating DS‐rTMS on post‐stroke upper limb function impairment demonstrated a similar, but significant, pooled effect size (0.56, *p* < 0.001), indicating that TMS was associated with significantly better improvement in upper limb function than tDCS (Figure 3). Due to the limited number of studies, other additional analysis was not possible.

### 3.5 Publication Bias

**FIGURE 4:**
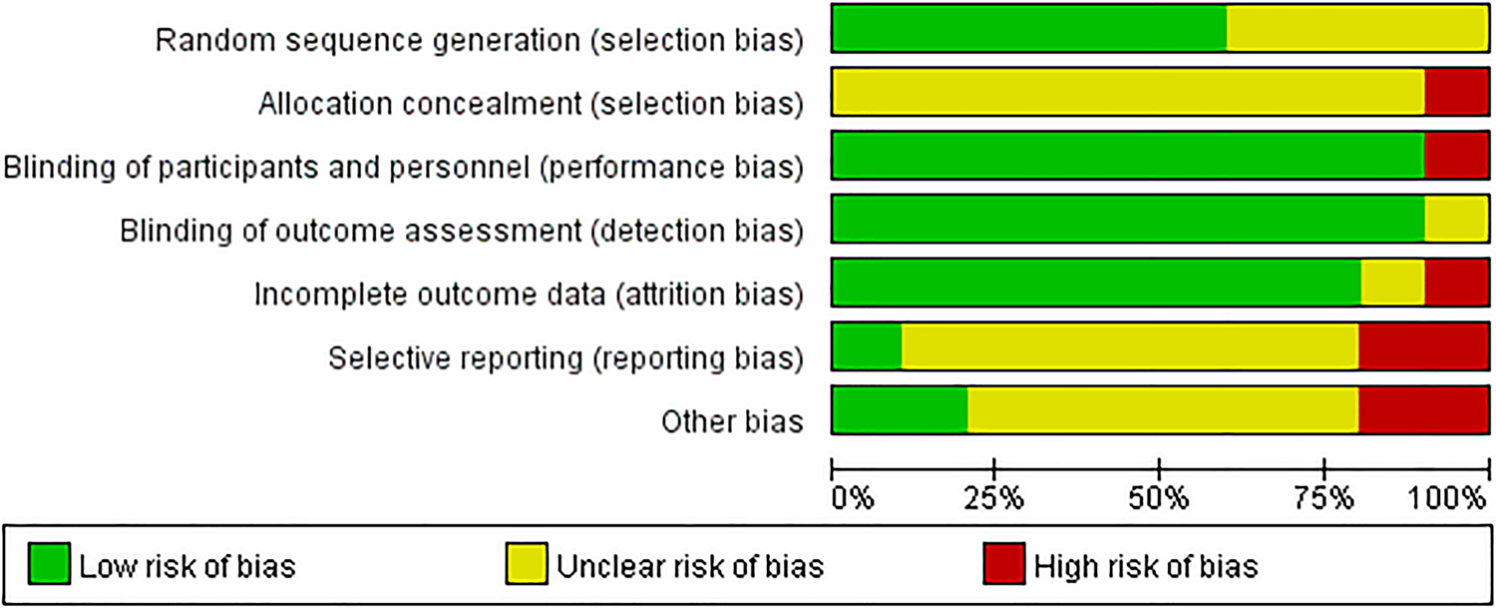
Results of the risk of bias analysis.

Figure 4 describes the total risk of bias in the 10 studies.

## 4 Discussion

The main purpose of this meta‐analysis was to analyze and summarize the current scientific literature in order to assess the efficacy of dual‐site NIBS on the upper limb motor impairments in post‐stroke patients. Our results show that dual‐site NIBS can yield improved upper limb function compared with sham and single‐site NIBS. This is the first meta‐analysis to report improvements in limb motor with DS‐rTMS compared to placebo stimulation, while no significant improvement was found with DS‐tDCS, suggesting DS‐rTMS may be superior to DS‐tDCS in improving upper limb motor after stroke.

Upper extremity motor impairment is a significant challenge in rehabilitation treatment after stroke. NIBS techniques are widely used to improve deficits following neuronal damage and have been reported to be successful in a proportion of treated patients (Davis and Koningsbruggen 2013). However, the choice of stimulation parameters and targets determines the effect of acupuncture therapy to a large extent. Some studies demonstrated high efficacy of dual‐site NIBS compared to single‐site NIBS (Achacheluee et al. 2018; Cho et al. 2017). As shown in Figures 2 and 3, our trial has yielded robust and consistent findings that support the benefits of dual‐site NIBS despite differences in NIBS types, post‐stroke duration, and treatment sessions.

One study (Taud et al. 2021) found no significant difference between DS‐tDCS and SS‐tDCS in facilitating recovery of upper extremity function. This result should be interpreted cautiously due to the small sample size and high inter‐individual variance in baseline motor function, lesion site, location and extent, time since stroke, age, and so on. Another study (Fleming et al. 2017) showed significant improvements in JTT performance after anodal or cathodal tDCS but not after bihemispheric stimulation. The reason why bihemispheric tDCS was ineffective is unknown. It might be due to differences in the structures that be stimulated and the changes in connectivity between brain regions relative to the unilateral arrangements. A meta‐analysis found that the linear response did not necessarily exist between session and tDCS effect; the effect of tDCS ≤ 10 sessions on upper limb function recovery in stroke patients was significantly higher than that of other sessions both with anode and cathode stimulation (Bai et al. 2019).

It is worth considering that all included studies in this meta‐analysis employ neuromodulation based on the inter‐hemispheric inhibitory competition model. The interhemispheric competition mechanism shows a reciprocal inhibition of the neural activity between bilateral hemispheres in a healthy brain, which is realized by the transcallosal fibers. After a unilateral stroke, the balance between the bilateral hemispheres of patients is broken, resulting in excessive excitation of the unaffected hemisphere and increased inhibition of the affected hemisphere (Bertolucci, Chisari, and Fregni 2018). The subsequent recovery is related to the connection of the brain network between the two hemispheres (Swayne et al. 2008). Therefore, rebalance of the brain is the key for the recovery of function (Tang et al. 2015). Bilateral NIBS is more conducive to achieving this balance. Nine of the ten included studies activate the affected hemisphere's M1 and suppress the unaffected hemisphere's M1, thereby correcting imbalanced interhemispheric competition. Only one study (Cao et al. 2022) considers the possible role of cerebella and activates the contralateral cerebellar cortex and iM1 (nonsimultaneous stimulation of contralateral cerebellar cortex before iM1). Some researchers found the interhemispheric imbalance resulting from stroke is time‐dependent, increasing in the early weeks after stroke. Sasaki et al. (Sasaki, Kakuda, and Abo 2014) compared a combined protocol of 10‐Hz ipsilesional rTMS and 1‐Hz contralesional rTMS with a single 10‐Hz ipsilesional rTMS in 58 patients (< 15 days poststroke). They found that the bilateral rTMS group showed significantly greater improvement in the Bruunstrom Recovery Stage than the 10‐Hz rTMS group. A meta‐analysis showed that both theta burst stimulation (TBS) and rTMS were found to be significantly more effective in the acute phase of stroke, but TBS was more effective than rTMS. However, rTMS was found to be more effective than TBS stimulation in patients in the subacute and chronic phases of stroke (Chen et al. 2022).

This meta‐analysis could not perform sub‐analyses to investigate effects of different post‐stroke duration and different stimulation parameters due to the limited number of studies and variability in stimulation parameters that have been reported, thus limiting our understanding of the positive changes in upper limb motor function promoted by DS‐NIBS.

In addition to the competition mechanism, a vicariation model and a bimodal balance‐recovery model have been proposed. The vicariation model suggests that the over‐activation of the contralesional hemisphere may be not a maladaptive but rather a vicarious mechanism through which the non‐lesioned hemisphere compensates for the affected one's functional and structural damage. The bimodal balance‐recovery model suggests that the mechanisms at the basis of an improvement after the lesion might change according to the amount of damage (Chen et al. 2023). The question as to whether the two hemispheres are in an inhibitory or facilitatory relationship and to what extent one mechanism takes over the other is still an open matter. However, there has been no research on DS‐NIBS for upper limb function impairment after stroke based on the other two models.

### 4.1 Limitations

Despite great efforts to minimize methodology differences across the selected studies, heterogeneity is yet unavoidable due to the large variability in the characteristics of patients across studies (chronic or acute, ischemic or hemorrhagic, cortical or subcortical lesion) and the lack of a standardized intervention protocol, making it difficult to reach a definite conclusion. Further, limited research on DS‐NIBS in the treatment of post‐stroke upper limb function impairment restricted further analysis.

## 5 Conclusion

DS‐NIBS showed a relatively higher effect than the sham and SS‐NIBS. In addition, DS‐rTMS demonstrated better therapeutic effects compared to DS‐tDCS on upper limb function impairment after stroke.

## Author Contributions

**Meng Ren**: writing–original draft, writing–review and editing, conceptualization. **Jingjing Xu**: writing–original draft, conceptualization. **Wenjing Wang**: software, visualization. **Lexian Shen**: writing–review & editing. **Chaojie Wang**: methodology, visualization. **Haoyang Liu**: data curation. **Lu Chen**: data curation. **Chanjing Liu**: data curation. **Yongheng Tang**: validation. **Tiantian Liu**: resources. **Jiening Wang**: resources.

## Conflicts of Interest

The authors declare no conflicts of interest.

### Peer Review

The peer review history for this article is available at https://publons.com/publon/10.1002/brb3.70145.

## Supporting information



Supporting Information

## Data Availability

The data that support the findings of this study are available from the corresponding author upon reasonable request.
