# Supplementary material for: Effect of Dual‐Site Non‐Invasive Brain Stimulation on Upper‐Limb Function After Stroke: A Systematic Review and Meta‐Analysis
Source: Brain Behav. 2024 Nov 7;14(11):e70145. doi: 10.1002/brb3.70145 (PMC11541860; doi:10.1002/brb3.70145)
Supplement: Supplementary file 1 — Supporting Information [file BRB3-14-e70145-s001.docx]

| **Database** | **Search Strategy** |
| --- | --- |
| MEDLINE  COCHRANE | ((("Stroke"[Mesh]) OR ((((((((((((((((((((((((((((Strokes[Title/Abstract]) OR (Cerebrovascular Accident[Title/Abstract])) OR (Cerebrovascular Accidents[Title/Abstract])) OR (CVA (Cerebrovascular Accident[Title/Abstract]))) OR (CVAs (Cerebrovascular Accident[Title/Abstract]))) OR (Cerebrovascular Apoplexy[Title/Abstract])) OR (Apoplexy, Cerebrovascular[Title/Abstract])) OR (Vascular Accident, Brain[Title/Abstract])) OR (Brain Vascular Accident[Title/Abstract])) OR (Brain Vascular Accidents[Title/Abstract])) OR (Vascular Accidents, Brain[Title/Abstract])) OR (Cerebrovascular Stroke[Title/Abstract])) OR (Cerebrovascular Strokes[Title/Abstract])) OR (Stroke, Cerebrovascular[Title/Abstract])) OR (Strokes, Cerebrovascular[Title/Abstract])) OR (Apoplexy[Title/Abstract])) OR (Cerebral Stroke[Title/Abstract])) OR (Cerebral Strokes[Title/Abstract])) OR (Stroke, Cerebral[Title/Abstract])) OR (Strokes, Cerebral[Title/Abstract])) OR (Stroke, Acute[Title/Abstract])) OR (Acute Stroke[Title/Abstract])) OR (Acute Strokes[Title/Abstract])) OR (Strokes, Acute[Title/Abstract])) OR (Cerebrovascular Accident, Acute[Title/Abstract])) OR (Acute Cerebrovascular Accident[Title/Abstract])) OR (Acute Cerebrovascular Accidents[Title/Abstract])) OR (Cerebrovascular Accidents, Acute[Title/Abstract]))) AND (("Upper Extremity"[Mesh]) OR ((((((((Extremities, Upper[Title/Abstract]) OR (Upper Extremities[Title/Abstract])) OR (Membrum superius[Title/Abstract])) OR (Upper Limb[Title/Abstract])) OR (Limb, Upper[Title/Abstract])) OR (Limbs, Upper[Title/Abstract])) OR (Upper Limbs[Title/Abstract])) OR (Extremity, Upper[Title/Abstract])))) AND (((("Transcranial Direct Current Stimulation"[Mesh]) OR ((((((((((((((((((((((((tDCS[Title/Abstract]) OR (Cathodal Stimulation Transcranial Direct Current Stimulation[Title/Abstract])) OR (Cathodal Stimulation tDCSs[Title/Abstract])) OR (Cathodal Stimulation tDCS[Title/Abstract])) OR (Stimulation tDCS, Cathodal[Title/Abstract])) OR (Stimulation tDCSs, Cathodal[Title/Abstract])) OR (tDCS, Cathodal Stimulation[Title/Abstract])) OR (tDCSs, Cathodal Stimulation[Title/Abstract])) OR (Transcranial Random Noise Stimulation[Title/Abstract])) OR (Transcranial Alternating Current Stimulation[Title/Abstract])) OR (Transcranial Electrical Stimulation[Title/Abstract])) OR (Electrical Stimulation, Transcranial[Title/Abstract])) OR (Electrical Stimulations, Transcranial[Title/Abstract])) OR (Stimulation, Transcranial Electrical[Title/Abstract])) OR (Stimulations, Transcranial Electrical[Title/Abstract])) OR (Transcranial Electrical Stimulations[Title/Abstract])) OR (Anodal Stimulation Transcranial Direct Current Stimulation[Title/Abstract])) OR (Anodal Stimulation tDCS[Title/Abstract])) OR (Anodal Stimulation tDCSs[Title/Abstract])) OR (Stimulation tDCS, Anodal[Title/Abstract])) OR (Stimulation tDCSs, Anodal[Title/Abstract])) OR (tDCS, Anodal Stimulation[Title/Abstract])) OR (tDCSs, Anodal Stimulation[Title/Abstract])) OR (Repetitive Transcranial Electrical Stimulation[Title/Abstract]))) OR ("Transcranial Magnetic Stimulation"[Mesh])) OR ((((((((Magnetic Stimulation, Transcranial[Title/Abstract]) OR (Magnetic Stimulations, Transcranial[Title/Abstract])) OR (Stimulation, Transcranial Magnetic[Title/Abstract])) OR (Stimulations, Transcranial Magnetic[Title/Abstract])) OR (Transcranial Magnetic Stimulations[Title/Abstract])) OR (Transcranial Magnetic Stimulation, Single Pulse[Title/Abstract])) OR (Transcranial Magnetic Stimulation, Paired Pulse[Title/Abstract])) OR (Transcranial Magnetic Stimulation, Repetitive[Title/Abstract]))) |
| CINAHAL EMBASE | #1.((stroke:ab,ti OR strokes:ab,ti OR 'cerebrovascular accident':ab,ti OR 'cerebrovascular accidents':ab,ti OR cva:ab,ti) AND 'cerebrovascular accident':ab,ti OR cvas:ab,ti) AND 'cerebrovascular accident':ab,ti AND 'or cerebrovascular apoplexy':ab,ti OR 'apoplexy, cerebrovascular':ab,ti OR 'vascular accident, brain':ab,ti OR 'brain vascular accident':ab,ti OR 'brain vascular accidents':ab,ti OR 'vascular accidents, brain':ab,ti OR 'cerebrovascular stroke':ab,ti OR 'cerebrovascular strokes':ab,ti OR 'stroke, cerebrovascular':ab,ti OR 'strokes, cerebrovascular':ab,ti OR apoplexy:ab,ti OR 'cerebral stroke':ab,ti OR 'cerebral strokes':ab,ti OR 'stroke, cerebral':ab,ti OR 'strokes, cerebral':ab,ti OR 'stroke, acute':ab,ti OR 'acute stroke':ab,ti OR 'acute strokes':ab,ti OR 'strokes, acute':ab,ti OR 'cerebrovascular accident, acute':ab,ti OR 'acute cerebrovascular accident':ab,ti OR 'acute cerebrovascular accidents':ab,ti OR 'cerebrovascular accidents, acute':ab,ti |
|  | #2.'transcranial direct current stimulation':ab,ti OR tdcs:ab,ti OR 'cathodal stimulation transcranial direct current stimulation':ab,ti OR 'cathodal stimulation tdcs':ab,ti OR 'cathodal stimulation tdcss':ab,ti OR 'stimulation tdcs, cathodal':ab,ti OR 'stimulation tdcss, cathodal':ab,ti OR 'tdcs, cathodal stimulation':ab,ti OR 'tdcss, cathodal stimulation':ab,ti OR 'transcranial random noise stimulation':ab,ti OR 'transcranial alternating current stimulation':ab,ti OR 'transcranial electrical stimulation':ab,ti OR 'electrical stimulation, transcranial':ab,ti OR 'electrical stimulations, transcranial':ab,ti OR 'stimulation, transcranial electrical':ab,ti OR 'stimulations, transcranial electrical':ab,ti OR 'transcranial electrical stimulations':ab,ti OR 'anodal stimulation transcranial direct current stimulation':ab,ti OR 'anodal stimulation tdcs':ab,ti OR 'anodal stimulation tdcss':ab,ti OR 'stimulation tdcs, anodal':ab,ti OR 'stimulation tdcss, anodal':ab,ti OR 'tdcs, anodal stimulation':ab,ti OR 'tdcss, anodal stimulation':ab,ti OR 'repetitive transcranial electrical stimulation':ab,ti OR 'transcranial magnetic stimulation':ab,ti OR 'magnetic stimulation, transcranial':ab,ti OR 'magnetic stimulations, transcranial':ab,ti OR 'stimulation, transcranial magnetic':ab,ti OR 'stimulations, transcranial magnetic':ab,ti OR 'transcranial magnetic stimulations':ab,ti OR 'transcranial magnetic stimulation, single pulse':ab,ti OR 'transcranial magnetic stimulation, paired pulse':ab,ti OR 'transcranial magnetic stimulation, repetitive':ab,ti |
|  | #3.'upper extremity':ab,ti OR 'extremities, upper':ab,ti OR 'upper extremities':ab,ti OR 'membrum superius':ab,ti OR 'upper limb':ab,ti OR 'limb, upper':ab,ti OR 'limbs, upper':ab,ti OR 'upper limbs':ab,ti OR 'extremity, upper':ab,ti |
|  | #4.#1 AND #2 AND #3 |
| WEB OF SCIENCE | TS=(Stroke OR Strokes OR Cerebrovascular Accident OR Cerebrovascular Accidents OR CVA (Cerebrovascular Accident) OR CVAs (Cerebrovascular Accident)OR Cerebrovascular Apoplexy OR Apoplexy, Cerebrovascular OR Vascular Accident, Brain OR Brain Vascular Accident OR Brain Vascular Accidents OR Vascular Accidents, Brain OR Cerebrovascular Stroke OR Cerebrovascular Strokes OR Stroke, Cerebrovascular OR Strokes, Cerebrovascular OR Apoplexy OR Cerebral Stroke OR Cerebral Strokes OR Stroke, Cerebral OR Strokes, Cerebral OR Stroke, Acute OR Acute Stroke OR Acute Strokes OR Strokes, Acute OR Cerebrovascular Accident, Acute OR Acute Cerebrovascular Accident OR Acute Cerebrovascular Accidents OR Cerebrovascular Accidents, Acute) AND TS=(Transcranial Direct Current Stimulation OR tDCS OR Cathodal Stimulation Transcranial Direct Current Stimulation OR Cathodal Stimulation tDCS OR Cathodal Stimulation tDCSs OR Stimulation tDCS, Cathodal OR Stimulation tDCSs, Cathodal OR tDCS, Cathodal Stimulation OR tDCSs, Cathodal Stimulation OR Transcranial Random Noise Stimulation OR Transcranial Alternating Current Stimulation OR Transcranial Electrical Stimulation OR Electrical Stimulation, Transcranial OR Electrical Stimulations, Transcranial OR Stimulation, Transcranial Electrical OR Stimulations, Transcranial Electrical OR Transcranial Electrical Stimulations OR Anodal Stimulation Transcranial Direct Current Stimulation OR Anodal Stimulation tDCS OR Anodal Stimulation tDCSs OR Stimulation tDCS, Anodal OR Stimulation tDCSs, Anodal OR tDCS, Anodal Stimulation OR tDCSs, Anodal Stimulation OR Repetitive Transcranial Electrical Stimulation OR Transcranial Magnetic Stimulation OR Magnetic Stimulation, Transcranial OR Magnetic Stimulations, Transcranial OR Stimulation, Transcranial Magnetic OR Stimulations, Transcranial Magnetic OR Transcranial Magnetic Stimulations OR Transcranial Magnetic Stimulation, Single Pulse OR Transcranial Magnetic Stimulation, Paired Pulse OR Transcranial Magnetic Stimulation, Repetitive) AND TS=(Upper Extremity OR Extremities, Upper OR Upper Extremities OR Membrum superius OR Upper Limb OR Limb, Upper OR Limbs, Upper OR Upper Limbs OR Extremity, Upper) AND TS=(Randomized Controlled Trial OR controlled clinical trial OR random allocation OR double-blind OR single-blind OR Placebo OR Randomly OR randomized OR clinical trial* OR trial* OR RCT OR Random) |
| 中国知网 | (主题:卒中（精确))OR(篇关摘:急性卒中＋脑卒中＋脑中风＋脑血管意外＋CVA + CVAs ＋脑血管中风＋中风，急性＋急性脑血管意外＋急性脑卒中(精确))AND ((主题:上肢（精确))OR(篇关摘:上肢（精确)） )AND ((主题:经颅直流电刺激（精确))OR(篇关摘:经颅直流电刺激＋经颅随机噪声刺激＋经颅交流电刺激＋经颅电刺激＋阳极经颅直流电刺激＋重复经颅电刺激＋阴极经颅直流电刺激（精确)）OR(主题:经颅磁刺激（精确)）OR(篇关摘:经颅磁刺激＋经颅磁刺激,单脉冲＋经颅磁刺激,成对脉冲＋经颅磁刺激,重复（精确)）) |
| 万方 | (主题:(卒中) or 题名或关键词:(急性卒中 or 脑卒中 or 脑中风 or 脑血管意外 or CVA or CVAs or 脑血管中风 or 中风，急性 or 急性脑血管意外 or 急性脑卒中)) and (主题:(上肢)) and (主题:(经颅直流电刺激 or 经颅磁刺激) or 题名或关键词:(经颅随机噪声刺激 or 经颅交流电刺激 or 经颅电刺激 or 阳极经颅直流电刺激 or 重复经颅电刺激 or 阴极经颅直流电刺激 or 经颅磁刺激, 单脉冲 or 经颅磁刺激, 成对脉冲 or 经颅磁刺激, 重复)) |
| 维普 | 题名或关键词=卒中or急性卒中or脑卒中or脑中风 or脑血管意外or CVA or CVAs or脑血管中风 or 中风，急性or急性脑血管意外or急性脑卒中AND题名或关键词=上肢AND题名或关键词=经颅直流电刺激or经颅随机噪声刺激or经颅交流电刺激or经颅电刺激or阳极经颅直流电刺激or重复经颅电刺激or阴极经颅直流电刺激or经颅磁刺激or经颅磁刺激,单脉冲or经颅磁刺激，成对脉冲or 经颅磁刺激,重复 |
| 中国生物医学文献数据库 | ("经颅直流电刺激"[常用字段:智能] OR "经颅随机噪声刺激"[常用字段:智能] OR "经颅交流电刺激"[常用字段:智能] AND "经颅电刺激"[常用字段:智能] OR "阳极经颅直流电刺激"[常用字段:智能] OR "重复经颅电刺激"[常用字段:智能] OR "阴极经颅直流电刺激"[常用字段:智能] AND "经颅磁刺激"[常用字段:智能]) AND ("上肢"[常用字段:智能]) AND ("卒中"[常用字段:智能] OR "急性卒中"[常用字段:智能] OR "脑卒中"[常用字段:智能] OR "脑中风"[常用字段:智能] OR "脑血管意外"[常用字段:智能] OR "CVA"[常用字段:智能] OR "CVAs"[常用字段:智能] OR "脑血管中风"[常用字段:智能]) |
